# Short- and long-term effects of dapagliflozin as an adjunct to insulin therapy in diabetic dogs: a pilot study

**DOI:** 10.1007/s11259-026-11604-7

**Published:** 2026-11-06

**Authors:** Taís Bock Nogueira, José Lucas Xavier Lopes, Frederico Aécio Carvalho Soares, Denise Iparraguirre da Silva, Caroline Bernardo Gusmão, Gabriela Goulart Teixeira, Álan Gomes Pöppl

**Affiliations:** 1 Veterinary Sciences Post-Graduation Program, Federal University of Rio Grande do Sul, Porto Alegre, Rio Grande do Sul Brazil; 2 Porto Alegre, Brazil; 3 Veterinary Faculty, Federal University of Rio Grande do Sul, Porto Alegre, Rio Grande do Sul Brazil; 4 Department of Animal Medicine, Federal University of Rio Grande do Sul, Porto Alegre, Rio Grande do Sul Brazil

**Keywords:** Sodium-glucose cotransporter 2, Oral hypoglycemic drug, Gliflozin, Canine

## Abstract

This study evaluated adjunctive dapagliflozin therapy in insulin-treated diabetic dogs to assess short- and long-term glycemic, clinical, and laboratory outcomes in dogs with naturally occurring diabetes mellitus. Six diabetic dogs received a compounded liquid oral dapagliflozin (CLODapa) formulation (0.088 ± 0.013 mg/kg once daily) in addition to ongoing insulin therapy for 100 days. We monitored continuous glucose monitoring (CGM) before and after treatment initiation, and serially assessed clinical, biochemical, and urinary parameters. Short-term dapagliflozin administration significantly reduced mean interstitial glucose, glucose nadir and peak values, and increased time within the target glucose range (60–250 mg/dL), while decreasing time above range. Glycemic variability increased, although time spent < 60 mg/dL and clinical hypoglycemia did not increase; however, CLODapa’s initial dosage was reduced in four dogs due to concerns about hypoglycemia, increased in one, and left unchanged in another. Long-term treatment was associated with reductions in fructosamine, systolic blood pressure, alanine aminotransferase, aspartate aminotransferase, alkaline phosphatase, and cholesterol concentrations. No evidence of diabetic ketoacidosis, urinary tract infection, electrolyte imbalance, or acid–base disturbance was detected. Unintentional weight loss was documented in three patients and required a ~ 25% increase in food intake. Adjunctive dapagliflozin showed promise in improving glycemic control in diabetic dogs despite posing a risk of hypoglycemia, supporting further investigation of SGLT2 inhibition in canine diabetology.

## Introduction

Virtually all diabetic dogs are insulin-deficient at diagnosis (Bugbee et al. 2026; Niessen et al. 2022) and require insulin therapy to control hyperglycemia, prevent diabetic ketoacidosis (DKA), and survive, resembling human type 1 diabetes mellitus (T1D) (Fleeman and Gilor 2023). Despite the use of modern insulin analogs (Tardo et al. 2024; Mott et al. 2025), continuous glucose monitoring (CGM) systems (Del Baldo et al. 2025), and commercial and home-made diabetic diets (Tardo et al. 2025), glycemic control in some diabetic dogs remains challenging (Bugbee et al. 2026; Kuzi et al. 2023), as also observed in humans (Mazzota et al. 2024). Strategies to improve glycemic control in human T1D, such as adjunct SGLT2 inhibitor (SGLT2i) therapy, remain controversial because they increase DKA risk; however, potential benefits are recognized (Maran et al. 2025).

Adjunctive SGLT2i therapy has been explored with enavogliflozin (An et al. 2024, 2025) and canagliflozin (Box et al. 2024) in diabetic dogs on insulin therapy and has been associated with benefits in glycemic control. Dapagliflozin as an add-on therapy (Dandona et al. 2018; Mazzota et al. 2024) was approved in some human T1D scenarios, and despite the description of its metabolic effects in a canine model of insulin resistance (Kabir et al. 2023) and some studies on the cardiovascular (Saengklub et al. 2025; Massey et al. 2026) and urinary (Pakhawala 2026; Umezawa et al. 2026) effects of this specific SGLT2i in dogs, its use in canine diabetes remains undocumented. Therefore, this pilot study aimed to characterize the short- and long-term impact of adding dapagliflozin to insulin therapy on CGM, laboratory, and clinical outcomes of naturally occurring diabetic dogs.

## Materials and methods

### Animals

This non-controlled clinical trial included six insulin-treated dogs with diabetes mellitus (DM): five spayed females (three Poodles, one Pinscher, one Shih-Tzu) and one neutered male Yorkshire Terrier. The median DM duration was 7 months (range 2–29), and the mean age was 9.83 ± 2.31 years. Owners signed an informed consent form prior to the dog´s enrollment in the study. The Ethics Committee on Animal Use in Research (CEUA/UFRGS) approved the study (protocol 48053). Insulin regimens were Glargine 300U q12h in three dogs, Glargine 100U q12h in one dog, NPH q12h in one dog, and a basal-bolus regimen with Glargine 300U + NPH q12h in one dog.

### Protocol

Dogs were evaluated at baseline (D0) and at 10 (D10), 40 (D40), and 100 (D100) days after dapagliflozin initiation. To enable dose titration, dapagliflozin was administered as a compounded liquid oral formulation (CLODapa) at 0.088 ± 0.013 mg/kg once daily, given at home by owners without changes to insulin therapy or feeding protocol.

Short-term evaluation assessed interstitial glucose (IG) metrics obtained with a CGM system (FreeStyle Libre 2, Abbott), previously validated in diabetic dogs (Del Baldo et al. 2025). The IG was monitored for a median of 5 days (range, 4–8) before initiating dapagliflozin and 13.5 days (11–20) after. Raw data were downloaded (.csv) from the manufacturer’s website (LibreView) to calculate mean IG, glycemic variability (GV%), and percentage of time (%T) < 60 mg/dL, within target range (60–250 mg/dL), and > 250 mg/dL. CLODapa dose adjustments were guided by individual clinical judgment based on the overall CGM data.

Long-term evaluations at each time point included insulin dose, physical examination, body weight, Diabetes Clinical Score (DCS) (Niessen et al. 2022), and systolic blood pressure measured by vascular Doppler according to Acierno et al. (2018). Laboratory assessments comprised complete blood count (CBC), serum biochemistry (albumin, beta-hydroxybutyrate (BHB), fructosamine, glucose, creatinine, SDMA, ALP, ALT, AST, phosphorus, cholesterol, triglycerides, urea, electrolytes [Na+, K+, iCa2+, Cl−]), venous blood gas analysis (VBGA), and urinalysis, including urinary protein-to-creatinine ratio (UPC).

All blood samples were collected after a 12-hour fast. CBC parameters were determined on an automated analyzer (Procyte DX, IDEXX), followed by manual differential counting. Serum biochemistry and UPC were evaluated on an automated analyzer (CM200, Wiener Lab). BHB was determined using a point-of-care ketometer (FreeStyle Optium Neo, Abbott). Electrolytes and VBGA were performed on an automated analyzer (Cobas b121, Roche). The insulin dose every 12 h was multiplied by fructosamine (mmol/L) to determine the insulin resistance index (Gostelow et al. 2017).

### Statistical analysis

Data distribution was assessed by the Shapiro-Wilk test. Paired t-tests or Wilcoxon signed-rank tests were used for pre- and post-CLODapa CGM comparisons according to parametric or non-parametric data distribution, respectively. Comparisons across time points were performed using repeated-measures ANOVA with Tukey’s post hoc test or the Friedman test with Dunn’s post hoc test, as appropriate. Analyses were conducted using GraphPad Prism 8.0.1. No primary outcome was set for this pilot study; therefore, the analyses should be interpreted as exploratory.

## Results

**Fig. 1:**
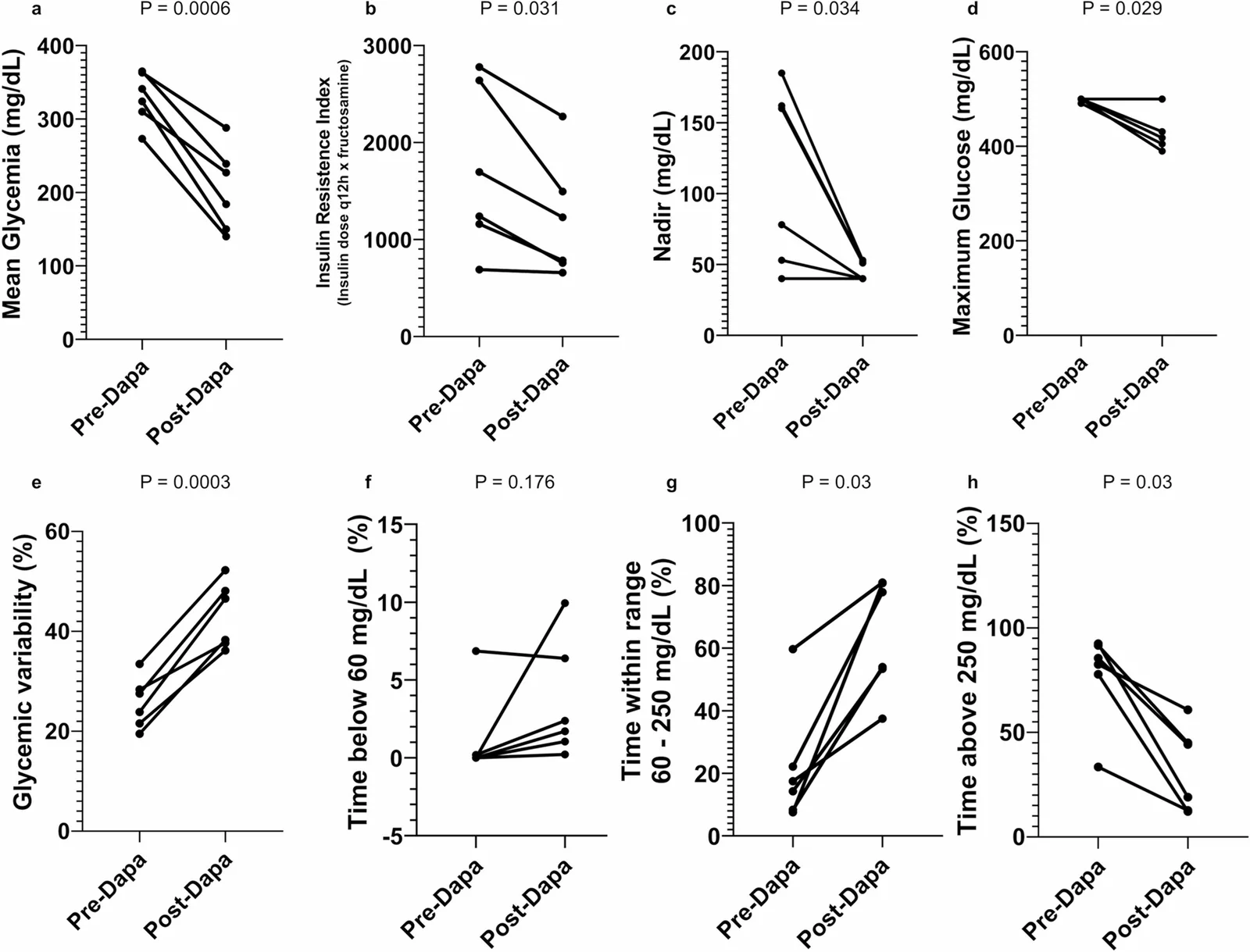
Short-term effects of dapagliflozin as an adjunctive therapy to insulin on continuous glucose monitoring (FreeStyle Libre 2) metrics in naturally occurring diabetic dogs. (**a**) Mean interstitial glucose. (**b**) Insulin resistance index (serum fructosamine x insulin dose q12h). (**c**) Lowest documented interstitial glucose (nadir). (**d**) Maximum documented interstitial glucose. (**e**) Glycemic variability (%). (**f**) Percentage of time with interstitial glucose below 60 mg/dL. (**g**) Percentage of time with interstitial glucose between 60 and 250 mg/dL. (**h**) Percentage of time with interstitial glucose above 250 mg/dL

Short-term analysis showed reduced mean post-CLODapa IG (*P* = 0.0006, Fig. 1a), insulin resistance index (*P* = 0.031, Fig. 1b), nadir glucose (*P* = 0.034, Fig. 1c), and maximum glucose (*P* = 0.029, Fig. 1d), whereas GV% increased (*P* = 0.0003, Fig. 1e). Time < 60 mg/dL was unchanged (*P* = 0.176, Fig. 1f), while time within 60–250 mg/dL increased (*P* = 0.03, Fig. 1g) and time > 250 mg/dL decreased (*P* = 0.03, Fig. 1h).

**Fig. 2:**
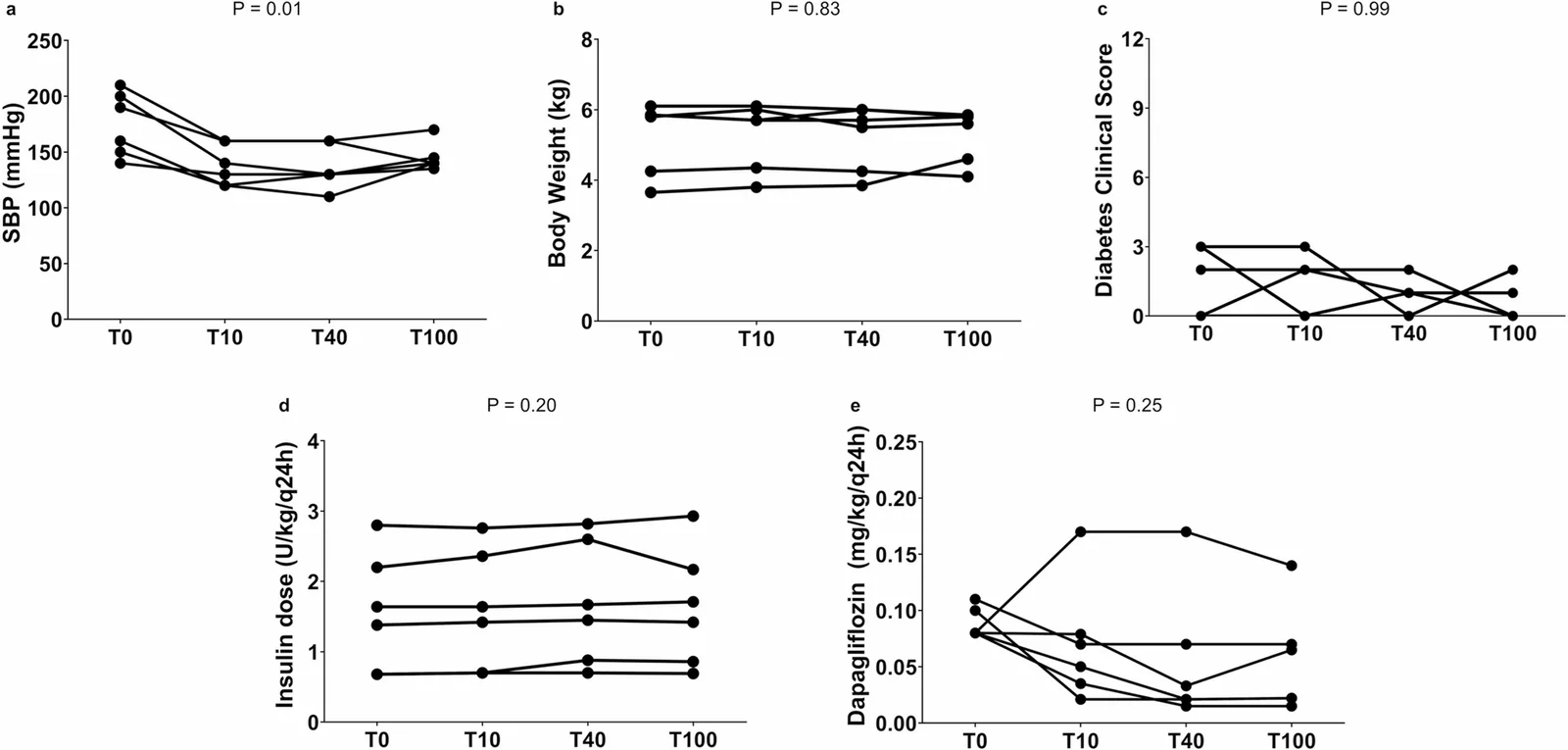
Long-term effects of dapagliflozin as an adjunctive therapy to insulin in naturally occurring diabetic dogs. (**a**) systolic blood pressure (SBP) (**b**) body weight (**c**) Diabetes Clinical Score. Figure 2d and e depict the daily insulin dose and the daily dapagliflozin dose over the studied period, respectively

Long-term evaluations showed reductions in ALT (*P* = 0.02), AST (*P* = 0.001), ALP (*P* = 0.04), total cholesterol (*P* = 0.01), fructosamine (*P* = 0.04), and systolic blood pressure (*P* = 0.01, Fig. 2a). No differences (*P* > 0.05) were observed in body weight (Fig. 2b), DCS (Fig. 2c), daily insulin dose (Fig. 2d), triglycerides, BHB, electrolytes, VBGA, proteinuria, or other evaluated variables (Table 1). No clinical hypoglycemia occurred, and no cataract progression was observed in the three patients without cataracts at enrollment. CLODapa dosage was reduced in four dogs due to concerns about low IG, increased in one, and remained unchanged in one (Fig. 2e). Three dogs experienced unintentional weight loss during the period studied and were managed with a ~ 25% increase in food intake.

**Table 1:** Selected parameters from the complete blood count, serum biochemistry, electrolytes, venous blood gas analysis, and urinalysis from six naturally occurring diabetic dogs on insulin therapy and monitored for 10 (T10), 40 (T40), and 100 (T100) days after initiating (T0) dapagliflozin as an adjunctive therapy

	T0	T10	T40	T100
Hematocrit (%)	47.8 ± 5.41	46.5 ± 4.63	44 ± 2.94	45.7 ± 2.94
Albumin (g/L)	33.2 ± 4.45	33.3 ± 3.61	32.5 ± 5.78	33.5 ± 4.03
**ALP (U/L)**	360.5 ± 172	**279.5 ± 124**^**a**^	**247.5 ± 103**^**a**^	**184.7 ± 39.1**^**a**^
**ALT (U/L)**	142.5 (49–269)	**93.5 (72–145)**^**a**^	**40 (12–106)**^**a**^	**54.5 (27–258)**^**a**^
**AST (U/L)**	40 ± 10.7	35 ± 9.52	**21.7 ± 10.7**^**b**^	34.8 ± 4.99
Creatinine (mg/dL)	0.6 ± 0.1	0.6 ± 0.21	0.58 ± 0.22	0.63 ± 0.18
Urea (mg/dL)	39.2 ± 10.38	44.33 ± 14.16	30.3 ± 9.71	40.7 ± 7.94
SDMA (µg/dL)	6.2 ± 2.23	7.2 ± 2.78	6.33 ± 0.81	6.5 ± 1.51
Phosphorus (mg/dL)	4.97 ± 1.11	4.88 ± 1.07	4.15 ± 0.93	4.7 ± 0.98
Glucose (mg/dL)	204.5 (57–537)	211.5 (48–325)	229.5 (49–346)	229.5 (172–345)
**Fructosamine (mmol/L)**	445.0 ± 120.3	**309.0 ± 69.61**^**a**^	**311.7 ± 97.48**^**a**^	**311.5 ± 97.2**^**a**^
Triglycerides (mg/dL)	133 (43–238)	179 (47–236)	76.5 (53–111)	118 (42–375)
**Cholesterol (mg/dL)**	294.8 ± 70.82	265.3 ± 85.22	**258 ± 56.13**^**c**^	268.3 ± 94.6
ß-OH-Butirate (mmol/L)	0.25 (0.1–0.3)	0.30 (0.2–1.2)	0.30 (0.2–1.2)	0.3 (0.3–0.7)
Na^++^ (mEq/L)	146.7 ± 4.47	146.6 ± 3.54	148.3 ± 4.69	146.2 ± 2.47
K^+^ (mEq/L)	4.24 ± 0.85	4.04 ± 0.39	4.06 ± 0.6	4.77 ± 0.38
Cl^−^ (mEq/L)	106.7 ± 3.84	108.7 ± 2.42	108.9 ± 2.65	106.6 ± 1.91
iCa^++^ (mEq/L)	1.27 ± 0.08	1.29 ± 0.03	1.25 ± 0.08	1.26 ± 0.04
pH (venous)	7.36 ± 0.03	7.35 ± 0.02	7.37 ± 0.03	7.37 ± 0.03
HCO_3_^−^ (mEq/L)	22.74 ± 2.04	22.46 ± 2.66	22.16 ± 2.89	22.96 ± 2.61
Sodium (mEq/L)	146.7 ± 4.47	146.6 ± 3.54	148.3 ± 4.69	146.2 ± 2.47
Specific urine gravity	1,038 ± 0,009	1,030 ± 0,015	1,038 ± 0,009	1,043 ± 0,006
Bacteriuria (+)	0 (0–2)	0.5 (0–2)	1 (0–2)	0 (0–2)
Ketonuria (+)	0 (0–0)	0 (0–0)	0 (0–0)	0 (0–0)
Glucosuria (+)	3 (0–4)	3.5 (2–4)	4 (3–4)	4 (2–4)
Urine protein: creatinine	0.1 (0.04–0.29)	0.08 (0.03–0.32)	0.06 (0.01–0.18)	0.05 (0.01–0.17)

^a^*p* < 0.05

^b^ Statistically different than T0 (*p* < 0.01) on Tukey’s multiple comparisons test

^c^ Statistically different than T0 (*p* < 0.05) on Tukey’s multiple comparisons test

Bold parameters showed significant variations in the long term

## Discussion

This study represents the first report of dapagliflozin combined with insulin therapy in dogs. The protocol improved short-term glycemic control by reducing mean CGM glucose, nadir, and maximum IG, while increasing time within range and decreasing time above range. The absence of clinical hypoglycemia, ketosis, and unchanged time < 60 mg/dL supports short-term safety regarding DKA risk, although monitoring for low IG values, β-hydroxybutyrate, and weight loss remains necessary. Beyond inhibiting renal glucose reabsorption, dapagliflozin induces metabolic adaptations in dogs, including lipolysis, adipose tissue remodeling, and adipokine modulation (Kabir et al. 2023), as well as ketogenic and diuretic effects (Umezawa et al. 2026). Unintentional weight loss required progressive increases in food intake (meal size) in half of the patients to prevent clinically relevant loss. Insulin-sparing and weight-loss effects were reported after one year of monitoring diabetic dogs on long-term insulin therapy exposed to enavogliflozin as adjunctive therapy (An et al. 2024). In contrast, a multicenter study of insulin-treated diabetic dogs on the same drug for eight weeks did not document weight loss (An et al. 2025), nor did another study evaluating short-term addition of canagliflozin to insulin therapy in diabetic dogs (Box et al. 2024).

The dapagliflozin dose previously reported in studies with non-diabetic dogs ranged from 0.3 to 1.25 mg/kg (Kabir et al. 2023; Saengklub et al. 2025; Massey et al. 2026), which were considered excessive for diabetic dogs on insulin therapy during the initial phase of this project, causing pronounced interstitial hypoglycemia and insulin-sparing effects. Yet, even with an initial dose of ~ 0.1 mg/kg in our study, most cases required reducing the CLODapa dose. Consistent with our findings, Umezawa et al. (2026) described increased 24-hour urinary glucose excretion in healthy dogs treated with 1 and 0.3 mg/kg compared with dogs receiving 0.1 mg/kg. The primary goal of adding dapagliflozin in these cases was to improve glycemic control, as shown by the CGM metrics. However, due to the insulin-deficient nature of most diabetic dogs (Bugbee et al. 2026), we did not specifically pursue insulin dose reduction because it can increase the risk of DKA and unintentional weight loss (Kabir et al. 2023; An et al. 2024; Maran et al. 2025). Despite minor individual adjustments, the mean patient insulin dose during the study remained unchanged. In contrast, although the mean CLODapa dose did not differ statistically over the study period, 66.7% of patients received dose reductions due to concerns about interstitial hypoglycemia, a therapy adjustment considered more appropriate than insulin dose reductions to avoid the above-mentioned risks. Some patients with laboratory hypoglycemia also showed BHB peaks (maximum 1.2 mmol/L) and a tendency toward weight loss despite no changes in insulin dosage, consistent with the metabolic effects of dapagliflozin described by Kabir et al. (2023). Although no dog had a DCS of 0/12 at enrollment, a score attributable to perfect clinical control (Niessen et al. 2022), it is important to emphasize that they had been on insulin therapy for months and were relatively well controlled based on the DCS. The main goals of diabetes therapy are to achieve control of clinical signs without inducing hypoglycemia (Niessen et al. 2022; Bugbee et al. 2026). In this way, further lowering glycemia with dapagliflozin in addition to insulin therapy in dogs with optimal clinical control may not result in clinical benefits and may carry a risk of hypoglycemia. These points highlight the importance of titrating dapagliflozin doses in diabetic dogs.

We hypothesized that dapagliflozin would improve glycemic control, potentially limit chronic diabetic complications, and support translational relevance to human T1D, while also serving as an alternative to improve glycemic control in dogs with challenging glycemic control. Although IG decreased, GV% increased, likely reflecting early insulin and CLODapa dose adjustments and lower baseline pre-CLODapa GV% at higher glycemic ranges. Cataract prevention is a desirable effect of tight glycemic control in dogs with diabetes, but the role of GV% in cataract induction in dogs remains unclear. Hyperglycemic dogs are more likely to need phacoemulsification surgery (PS), and increased GV% was positively correlated with PS indication in a small cohort of diabetic dogs (Ared et al. 2025); however, average glucose and fructosamine were also higher in this subset. The three patients without cataracts at enrollment did not show cataract progression, likely due to better glycemic control, despite increased GV% in the short term. Indeed, the improvement in the insulin resistance index should be interpreted as a marker of better glycemic control, rather than a direct improvement in insulin sensitivity, because it is calculated based on insulin dose and serum fructosamine concentration.

Adjunctive dapagliflozin showed promise for improving glycemic control in diabetic dogs and was associated with long-term reductions in systolic blood pressure, fructosamine, and liver enzymes. However, the small sample size may have reduced statistical power and increased the risk of type II error, whereas multiple comparisons may have increased the risk of type I error. Thus, appropriate caution is recommended when interpreting statistically significant findings given the study´s exploratory nature. CLODapa was well tolerated, with no evidence of urinary tract infection or electrolyte or acid–base disturbances, although monitoring for weight loss, hypoglycemia, and ketosis is highly recommended. Limitations of this pilot study, in addition to the small sample size, include heterogeneity of canine diabetes mellitus, variable insulin protocols, and the fact that enrolled dogs were relatively well controlled at enrolment, limiting documentation of the clinical benefits of the adjunctive therapy. Moreover, the long-term effect of the adjunctive therapy on GV% was not documented; nevertheless, these findings support further investigation in this translational field and in veterinary practice.

## Data Availability

The datasets generated during and/or analyzed during the current study are available from the corresponding author upon reasonable request.
